# Simultaneous Automated Screening and Confirmatory Testing for Vasculitis-Specific ANCA

**DOI:** 10.1371/journal.pone.0107743

**Published:** 2014-09-16

**Authors:** Mandy Sowa, Kai Grossmann, Ilka Knütter, Rico Hiemann, Nadja Röber, Ursula Anderer, Elena Csernok, Dimitrios P. Bogdanos, Maria Orietta Borghi, Pier Luigi Meroni, Peter Schierack, Dirk Reinhold, Karsten Conrad, Dirk Roggenbuck

**Affiliations:** 1 Research and Development Department, GA Generic Assays GmbH, Dahlewitz/Berlin, Germany; 2 Faculty of Science, Brandenburg University of Technology Cottbus-Senftenberg, Senftenberg, Germany; 3 Institute of Immunology, Technical University of Dresden, Dresden, Germany; 4 Department of Rheumatology, University of Schleswig-Holstein Campus Lübeck and Rheumaklinik Bad Bramstedt, Bad Bramstedt, Germany; 5 Division of Transplantation, Immunology and Mucosal Biology, King's University College, London, United Kingdom; 6 Department of Rheumatology, University of Milan, Milan, Italy; 7 Institute of Molecular and Clinical Immunology, Otto-von-Guericke University Magdeburg, Magdeburg, Germany; University of Michigan Medical School, United States of America

## Abstract

Anti-neutrophil cytoplasmic antibodies (ANCA) are the serological hallmark of small vessel vasculitis, so called ANCA-associated vasculitis. The international consensus requires testing by indirect immunofluorescence (IIF) on human ethanol-fixed neutrophils (ethN) as screening followed by confirmation with enzyme-linked immunosorbent assays (ELISAs). This study evaluates the combination of cell- and microbead-based digital IIF analysis of ANCA in one reaction environment by the novel multiplexing CytoBead technology for simultaneous screening and confirmatory ANCA testing. Sera of 592 individuals including 118 patients with ANCA-associated vasculitis, 133 with rheumatoid arthritis, 49 with infectious diseases, 77 with inflammatory bowel syndrome, 20 with autoimmune liver diseases, 70 with primary sclerosing cholangitis and 125 blood donors were tested for cytoplasmic ANCA (C-ANCA) and perinuclear ANCA (P-ANCA) by classical IIF and ANCA to proteinase 3 (PR3) and myeloperoxidase (MPO) by ELISA. These findings were compared to respective ANCA results determined by automated multiplex CytoBead technology using ethN and antigen-coated microbeads for microbead immunoassays. There was a good agreement for PR3- and MPO-ANCA and a very good one for P-ANCA and C-ANCA by classical and multiplex analysis (Cohen's kappa [κ] = 0.775, 0.720, 0.876, 0.820, respectively). The differences between classical testing and CytoBead analysis were not significant for PR3-ANCA, P-ANCA, and C-ANCA (p<0.05, respectively). The prevalence of confirmed positive ANCA findings by classical testing (IIF and ELISA) compared with multiplex CytoBead analysis (IIF and microbead immunoassay positive) resulted in a very good agreement (κ = 0.831) with no significant difference of both methods (p = 0.735). Automated endpoint-ANCA titer detection in one dilution demonstrated a very good agreement with classical analysis requiring dilution of samples (κ = 0.985). Multiplexing by CytoBead technology can be employed for simultaneous screening and quantitative confirmation of ANCA. This novel technique provides fast and cost-effective ANCA analysis by automated digital IIF for the first time.

## Introduction

Autoimmune vascular disorders comprising granulomatosis with polyangiitis (GPA, formerly Wegener's granulomatosis), microscopic polyangiitis (MPA), and eosinophilic granulomatosis with polyangiitis (EGPA, formerly Churg-Strauss syndrome) are characterized by microvascular inflammation, tissue necrosis, and the appearance of anti-neutrophil cytoplasmic antibody (ANCA) [1]–[6]. Thus, the term ANCA-associated vasculitis has been coined for this distinct disease group characterized by loss of tolerance to neutrophilic targets. According to the international consensus statement for ANCA testing, indirect immunofluorescence (IIF) findings on ethanol-fixed human neutrophils (ethN) are recommended to be confirmed with antigen-specific enzyme-linked immunosorbent assays (ELISAs) [2], [4], [7], [8]. ANCA IIF reveals two main patterns on ethN sub-classifying ANCAs into cytoplasmic ANCA (C-ANCA) and perinuclear ANCA (P-ANCA). The C- and P-ANCA in human patients with ANCA-associated vasculitis are mainly directed against proteinase 3 (PR3) and myeloperoxidase (MPO), respectively, and seem to be associated with disease activity [9], [10]. However, ANCA IIF patterns as well as PR3- and MPO-ANCA can be observed in other inflammatory conditions and several ANCA-specific targets apart from MPO and PR3 have been reported which lowers the specificity of ANCA testing by IIF [11],[12]. Thus, a C-ANCA pattern confirmed by PR3-ANCA ELISA positivity is indicative for GPA [1], [3], whereas a P-ANCA pattern confirmed by a positive MPO-ANCA ELISA finding supports the diagnosis of MPA and EGPA [11]. Furthermore, the corresponding ANCA titers are often associated with activity of disease in patients with GPA and MPA.

Consequently, appropriate ANCA testing requires two independent assay techniques to be run currently. Thus, the combination of both IIF and antigen-specific assays was confirmed in several studies to be the optimal strategy for ANCA detection [13].

Recently, IIF microscopy employing fluorescent microbeads as solid phase has been reported offering the opportunity to multiplex autoantibody analysis [14], [15]. For the first time, we employed this novel multiplexing technique along with ethN-based IIF for the development of one reaction environment to combine screening and confirmatory ANCA testing. Thus, pattern recognition of P-ANCA and C-ANCA on ethN was aligned with the quantitative determination of PR3- and MPO-ANCA by the means of a novel software module for the automated pattern recognition system Aklides. Existing multiplex ANCA testing such as the mosaic technique does not offer these benefits [16].

Automated digital IIF has been used in HEp2-cell based assays for analysis of antinuclear (ANA) and dsDNA antibodies. Moreover, analysis of respective autoantibody endpoint titers without serial dilution became available by the introduction of calibration tools for digital immunofluorescence [17]–[22]. We developed a similar technique for ANCA-endpoint titer determination by the novel combined ANCA test. Thus, the novel CytoBead test system presents a unique combination of a classical cell-based assay with multiplexing microbead technology for the simultaneous quantitative analysis of ANCA and their specificities to PR3 and MPO.

In the present study, we evaluated the performance of the novel CytoBead ANCA assay and compared it with classical ANCA testing by independent techniques. Furthermore, we compared the quantitative assessment of PR3- and MPO-ANCA as well as the ANCA-endpoint-titer analysis of the CytoBead ANCA assay on the automated interpretation system Aklides with classical ELISA and IIF methods.

## Materials and Methods

### Patients and controls

Sera of 592 individuals including 118 patients with ANCA-associated vasculitis, 300 with autoimmune and gastrointestinal diseases, 49 with infectious diseases, and 125 blood donors (BD) were enrolled for the present evaluation (Table 1; patient sera are non-consecutive). Patients with ANCA-associated vasculitis were diagnosed based on typical disease history, characteristic clinical findings, and confirmed clinical histology according to the criteria of the 1994 Chapel Hill Consensus Conference, the consensus statement of 1999, 2012 and the 1990 American College of Rheumatology [2], [4], [23]. Serum samples were obtained from patients with a confirmed clinical diagnosis of GPA or MPA irrespective of serology.

**Table 1:** Characteristics of patients and controls: 118 patients with anti-neutrophil cytoplasmic antibody (ANCA)-associated vasculitis, 300 with autoimmune and gastrointestinal diseases, 49 with infectious disorders, and 125 blood donors (BD) were enrolled in the study.

Diagnosis	N	Gender	Age	Age
		f/m	median	interquartile range
**ANCA-associated vasculitis**				
GPA	90	51/39	65	56–89
MPA	28	14/14	67	51–72
**Autoimmune disease controls**				
RA	133	99/34	62	56–69
PSC	70	21/49	45	35–57
AIH I	10	8/2	13	12–15
AIH II	10	10/0	11	8–14
UC	57	31/26	49	38–57
CD	20	15/5	40	32–54
**Infectious disease controls**				
Toxoplasmosis	16	15/1	34	27–43
CMV	25	23/2	38	33–41
Rubella	5	5/0	36	31–37
EBV	3	2/1	10	7–23
**Blood Donors**	125	64/61	21	21–26

AIH, autoimmune hepatitis; CMV, Cytomegalovirus; CD, Crohn's disease; EBV, Epstein-Barr virus; f, female; GPA, granulomatosis with polyangiitis; m, male; MPA, microscopic polyangiits; N, number; PSC, primary sclerosing cholangitis; RA, rheumatoid arthritis; UC, ulcerative colitis.

Serum samples from patients with rheumatoid arthritis (RA), primary sclerosing cholangitis (PSC), autoimmune hepatitis type1 and 2, ulcerative colitis (UC), Crohn's disease (CD) were used as disease controls (Table 1). In total, 49 sera from patients with infectious disease (cytomegalovirus [CMV], rubella virus, Toxoplasma gondii, Epstein-Barr virus [EBV]) were included as further disease controls. In particular, patients with herpes viral infections have the potential to induce ANCA production due to overall B cell stimulation and, thus, could demonstrate false-positive results.

The study received approval from the ethical committee of the Technical University of Dresden (EK56022014) and fulfilled the ethical guidelines of the most recent declaration of Helsinki. An approval of the donors was not necessary because fully anonymized probes used as quality controls in routine diagnostics were selected for this study only. The ethical committee waived the need for written informed consent from the participants accordingly.

### Detection of PR3- and MPO-ANCA with antigen-specific ELISA

PR3- and MPO-ANCA were detected using commercially available antigen-specific ELISAs according to instructions of the manufacturers (GA Generic Assays GmbH, Dahlewitz, Germany; Orgentec GmbH, Wiesbaden, Germany) as described elsewhere [24], [25]. The PR3- and MPO-ANCA ELISAs of GA Generic Assays GmbH revealed intra-assay variabilities of 5.2% each and inter-assay variabilities of 6.2% each for a serum with 20.0 U/mL PR3-ANCA and 20.0 U/mL MPO-ANCA, respectively. The PR3- and MPO-ANCA ELISAs of Orgentec GmbH revealed intra-assay variabilities of 3.3% and 4.1% for sera with 14.0 U/mL PR3-ANCA and 30.2 U/mL MPO-ANCA and inter-assay variabilities of 6.8% and 4.9% for sera with 51.7 U/mL PR3-ANCA and 33.8 U/mL MPO-ANCA, respectively.

### Determination of ANCA by indirect immunofluorescence

PR3- and MPO-ANCA have been analyzed by IIF employing a commercial kit with ethN (GA Generic Assays GmbH). Patient sera and control sera were diluted 1/20 and 50 µl per well were used. The sera were incubated for 30 minutes on the slides and afterwards washed five times each two minutes with phosphate buffered saline (PBS). Subsequently an AlexaFluor 488 conjugated polyclonal anti-human IgG antibody (Dianova GmbH, Hamburg, Germany) was used as secondary antibody and incubated again 30 minutes. After incubation the slides were washed accordingly and the wells were covered with a specific covering solution. The slides were evaluated automatically using the Aklides platform (Medipan, Berlin/Dahlewitz, Germany) as described elsewhere [26]. Briefly, images were assessed automatically using a motorized inverse microscope (IX81, Olympus Corporation, Tokyo, Japan) with a motorized scanning stage (IM120, Märzhäuser, Wetzlar, Germany); 400 nm and 490 nm light-emitting diodes (LED) (PrecisExcite, CoolLED, Andover, UK), and a charge-coupled device grey-scale camera (DX4, Kappa, Gleichen, Germany). The interpretation system is controlled by the Aklides software consisting of modules for device and autofocus control, image analysis, and pattern recognition algorithms. The novel autofocus based on Haralick's image characterization of objects through grey-scale transition using DAPI as fluorescent dye for focusing, quality evaluation, and object recognition. Two-dimensional images were acquired using an objective with 40-fold magnification (Olympus semiapochromat LUCPLFLN 40X, 0.60 NA, W.D. 2.7–4.0 mm). Fluorescence detection was performed using LED excitation with appropriate multiband filter for the DAPI and FITC dyes (DA/FI-A, Semrock, Rochester, USA). Single DAPI and FITC image were serially captured and stored in lossless compressed Tagged Image File (TIF) format.

### Multiplex detection of ANCA by CytoBead ANCA

**Figure 1:**
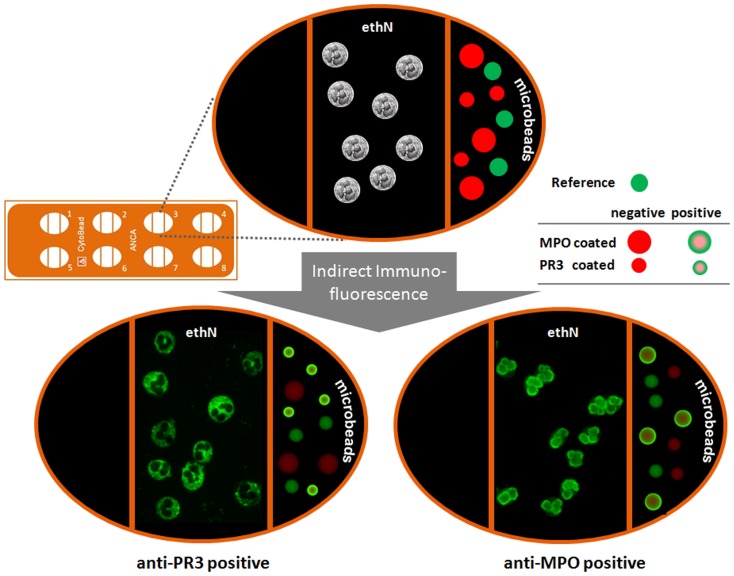
CytoBead ANCA assay principle. Microscopic glass slides with ethanol-fixed human neutrophils (ethN; middle compartment of the well) and proteinase 3 (PR3) as well as myeloperoxidase (MPO) coated microbeads (right compartment of the well) are used for detection of anti-neutrophil cytoplasmic antibodies (ANCAs) by ethN-based indirect immunofluorescence and simultaneous analysis of PR3- and MPO-ANCA by microbead immunoassay. PR3-ANCA positive sera show cytoplasmic fluorescence patterns on ethN and a green fluorescence halo on the surface of PR3-coated microbeads (9 µm). In contrast, MPO-ANCA positive sera show perinuclear fluorescence patterns on ethN and a green fluorescence halo on the surface of MPO-coated microbeads (15 µm).

CytoBead ANCA (GA Generic Assays GmbH) is a multiplex IIF test in one reaction environment combining the screening of ANCA on ethN and their confirmation with multiplex microbead immunoassays, using 9 µm and 15 µm red fluorescent microbeads (excitation 610 nm/emission 690 nm) coated with recombinant antigens PR3 and MPO, respectively. Triple parted wells on microscopic glass slides were employed for the fixation of neutrophils in the middle compartment as well as PR3- and MPO-coated microbeads in the right compartment (Fig. 1). The left compartment was not used and can be employed for further antibody determinations in the framework of an autoantibody profiling [27], [28]. Furthermore, a reference microbead population of 12 µm labelled by a green emitting fluorescence dye filling the entire microbead is immobilized on the right compartment. Thus, the differently sized green fluorescence halos of positively stained PR3- and MPO-coated microbeads can be distinguished. In general, PR3-ANCA positive sera show cytoplasmic fluorescence patterns on ethN and a green fluorescence halo on the surface of PR3-coated microbeads only. In contrast, MPO-ANCA positive sera show perinuclear fluorescence patterns on ethN and a green fluorescence halo on the surface of MPO-coated microbeads. For automation, the fluorescence intensities of the fluorescence halos can be quantified and simultaneously located to the appropriate microbead population by the Aklides system.

### Fully automated interpretation and pattern recognition of ANCA

The concept of the fully automated interpretation system Aklides for evaluation of ANCA IIF patterns is based on novel mathematical software algorithms for pattern recognition [25], [26]. To obtain a reproducible IIF read out signal, the excitation light intensity was calibrated employing a recently developed calibration tool [20]. Novel fluorescent calibration microbeads employed guarantee satisfactory inter-laboratory reproducibility for the calibration process.

Cells and microbeads were characterized by regional, topological, and texture/surface descriptors by employing image data of DAPI and FITC for cells and Cy5 for microbeads. A minimum of 20 stained ethN and 50 microbeads were counted at each sample. The obtained mean fluorescence intensities (MFI) reflect the specific ANCA reactivity of the serum sample. The final read out is expressed as arbitrary units.

### Automated endpoint-titer ANCA determination with Aklides

Automated ANCA endpoint-titer determination avoiding serial dilution of samples was developed using analysis algorithms for endpoint-titers of antinuclear antibodies described recently. In order to compensate the different MFI of the two ANCA patterns, the Aklides software harmonizes the fluorescence intensity measurement by including several object description characteristics for MFI analysis. Nevertheless, ANCA positive sera with differing classical end-point titers diluted at 1 to 20 revealed differing MFI values in particular for higher titers depending on the ANCA pattern. Thus, the novel software module for ANCA end-point titer determination employs different algorithms depending on the ANCA IIF pattern analysed by the Aklides system.

### Quantification of ANCA with lot-specific standard curves

For the quantification of ANCA by microbead immunoassays of CytoBead testing, computer-stored lot-specific standard master curves were established. Stable microbead reactivity permitted the use of a single lot-specific standard curve to quantify ANCA concentrations. Thus, these standard master curves were obtained by assaying dilutions (1/20 to 1/2560) of reference sera for MPO-ANCA (human reference serum #15) and PR3-ANCA (human reference serum #16) of the International Center for Disease Control and Prevention (CDC).

After acquisition by the Aklides system, PR3- and MPO-ANCA standard curves were fitted using a 5-parameter logistic-fitting curve model [29]. Curve fit parameters were then stored in a post-analysis charge certificate and provided for each assay run to analyze the obtained MFI data. Quantitative data are processed after recalibration of initial stored lot-specific master curves by a two-point recalibration using adjuster signal levels of the current run.

For assay performance assessment, intra- and inter-assay coefficients of variations (CV) were calculated by a eight-fold measurement of serum samples within one run (intra-assay) and further by measurement on 3 different days (inter-assay). The functional assay sensitivity (limit of quantification), being the lowest detectable concentration with an inter-assay CV lower or equal than 20%, for PR3- and MPO-ANCA was determined as described previously [30].

### Data standardisation

For the data comparison, ELISA findings in units per millilitre (U/ml) and CytoBead ANCA assay data in international units (IU) were standardized. The highest standard curve concentration points of the ELISA and CytoBead ANCA assay were referred to as 100% and results converted respectively.

### Statistical analysis

Inter-rater agreement statistics (Cohen's kappa, κ) and McNemar's test were used for group comparison. P values below 0.05 were considered to be significant. Receiver operating characteristics (ROC) curve analysis was performed using MedCalc software (MedCalc, Mariakerke, Belgium; Version 12.4.0).

## Results

### CytoBead ANCA cut-off determination

**Figure 2:**
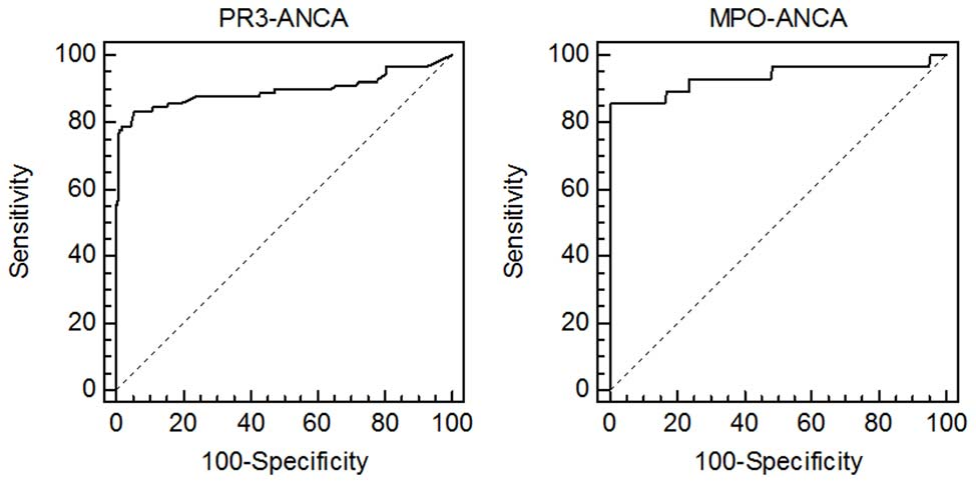
Receiver-operating characteristic curve analysis of anti-neutrophil cytoplasmic antibodies (ANCAs) to proteinase 3 (PR3) and myeloperoxidase (MPO) by CytoBead ANCA. 465 sera from 118 patients with ANCA-associated vasculitis, 133 with rheumatoid arthritis, 49 with infectious diseases, 20 with Crohn's disease, 20 with autoimmune hepatitis and 125 blood donors were included. PR3- and MPO-ANCA were determined simultaneously by microbead immunoassay employing 90 patients with granulomatosis with polyangiitis and 28 patients with microscopic polyangiitis as positive criterion, respectively.

To determine the cut-off of the novel CytoBead ANCA for PR3- and MPO-ANCA, 465 human sera of patients and controls including 118 patients with ANCA-associated vasculitis, 133 with RA, 49 with ID, 20 with CD, 20 with AIH and 125 BD were run with the Aklides IIF interpretation system. Patients with PSC and UC were excluded from the ROC curve analysis due to the known frequent number of positive ANCA (especially PR3-ANCA) findings in these patient groups. The obtained MFIs were standardised as described in Material and Methods and subjected to ROC curve analysis to obtain the respective cut-off values for each ANCA specificity (Fig. 2). For PR3-ANCA the calculated cut-off was 8.4% (0.9 IU/mL) and for MPO-ANCA 19.3% (3.0 IU/mL). The area under curve (AUC) was determined for PR3-ANCA employing 90 sera of patients with GPA as positive criterion at 0.896 (95% confidence interval [CI]: 0.864–0.923) and for MPO-ANCA using 28 patients with MPA as positive criterion at 0.934 (95% CI: 0.904–0.957); p<0.0001, respectively.

The PR3- and MPO-ANCA microbead immunoassays of the multiplex CytoBead testing displayed an intra-assay variability of 7.1% and 7.7% and an inter-assay variability of 7.2% and 7.6% for sera with 25 IU/mL PR3-ANCA and 100 IU/mL MPO-ANCA, respectively.

The functional assay sensitivity for PR3-ANCA and MPO-ANCA were analyzed as 5.8% (0.6 IU/ml) and 16.1% (2.5 IU/ml), respectively.

### Comparison of ANCA prevalences determined by classical and multiplex CytoBead assays

ANCA immunofluorescence pattern as well as PR3- and MPO-ANCA were determined by classical ELISA and IIF and compared with respective findings by automated IIF and microbead immunoassay employing the CytoBead technology (Table 2). According to inter-rater agreement statistics, there was a good agreement for PR3- and MPO-ANCA (κ = 0.775, 95% CI: 0.710–0.839; 0.720, 95% CI: 0.596–0.843, respectively, Table 3). The agreement for P-ANCA and C-ANCA between classical IIF and CytoBead analysis was very good (κ = 0.876, 95% CI: 0.812–0.940; 0.820, 95% CI: 0.755–0.844, respectively).

**Table 2:** Assessment of anti-neutrophil cytoplasmic antibodies (ANCA) and ANCA against proteinase 3 (PR3) and myeloperoxidase (MPO) by classical and automated multiplex analysis in one reaction environment.

	Classical testing					CytoBead assay				
	*ELISA*		*IIF*	*ELISA or IIF*	*ELISA and IIF*	*MIA*		*IIF*	*MIA or IIF*	*MIA and IIF*
samples (N)	*PR3-ANCA*	*MPO-ANCA*	*C/P-ANCA*			*PR3-ANCA*	*MPO-ANCA*	*C/P-ANCA*		
**GPA** (90)	78 (86.7%)	3* (3.3%)	77 (85.6%)	81 (90.0%)	77 (85.6%)	70 (77.8%)	11* (12.2%)	83 (92.2%)	85 (94.4%)	72 (80.0%)
**MPA** (28)	0 (0.0%)	20 (71.4%)	26 (92.9%)	26 (92.9%)	20 (71.4%)	0 (0.0%)	24 (85.7%)	28 (100.0%)	28 (100.0%)	24 (85.7%)
**RA** (133)	0 (0.0%)	0 (0.0%)	47 (35.3%)	47 (35.3%)	0 (0.0%)	0 (0.0%)	0 (0.0%)	47 (35.3%)	47 (35.3%)	0 (0.0%)
**PSC** (70)	31 (44.3%)	3 (4.3%)	49 (70.0%)	53 (75.7%)	27 (38.6%)	24 (34.3%)	1 (1.4%)	49 (70.0%)	52 (74.3%)	21 (30.0%)
**AIH1** (10)	3 (30.0%)	1 (10.0%)	9 (90.0%)	9 (90.0%)	1 (10.0%)	1 (10.0%)	1 (10.0%)	9 (90.0%)	9 (90.0%)	2 (20.0%)
**AIH2** (10)	1 (10.0%)	1 (10.0%)	3 (30.0%)	3 (30.0%)	2 (20.0%)	2 (20.0%)	1 (10.0%)	3 (30.0%)	3 (30.0%)	3 (30.0%)
**UC** (57)	10 (17.5%)	1 (1.8%)	26 (45.6%)	29 (50.9%)	8 (14.0%)	18 (31.6%)	1 (1.8%)	26 (45.6%)	33 (57.9%)	10 (17.5%)
**CD** (20)	0 (0.0%)	0 (0.0%)	2 (10.0%)	2 (10.0%)	0 (0.0%)	0 (0.0%)	0 (0.0%)	2 (10.0%)	2 (10.0%)	0 (0.0%)
**ID** (49)	0 (0.0%)	0 (0.0%)	0 (0.0%)	0 (0.0%)	0 (0.0%)	0 (0.0%)	0 (0.0%)	0 (0.0%)	0 (0.0%)	0 (0.0%)
**BD** (125)	0 (0.0%)	0 (0.0%)	7 (5.6%)	7 (5.6%)	0 (0.0%)	0 (0.0%)	0 (0.0%)	13 (10.4%)	13 (10.4%)	0 (0.0%)

ANCA were determined by enzyme-linked immunosorbent assay (ELISA), classical indirect immunofluorescence (IIF) on ethanol-fixed neutrophils and multiplex CytoBead assay in patients and controls: 118 patients with ANCA-associated vasculitis, 300 with autoimmune and gastrointestinal diseases, 49 with infectious disorders, and 125 blood donors (BD).

* p<0.05.

AIH, autoimmune hepatitis; BD, blood donors; CD, Crohn's disease; c/p ANCA, cytoplasmic/perinuclear ANCA; GPA, granulomatosis with polyangiitis; ID, infectious diseases; MIA, microbead immunoassay; MPA, microscopic polyangiitis; N, number; PSC, primary sclerosing cholangitis; RA, rheumatoid arthritis; UC, ulcerative colitis.

**Table 3:** Comparison of perinuclear (P-ANCA) and cytoplasmic (C-ANCA) anti-neutrophil cytoplasmic antibodies and ANCA against proteinase 3 (PR3) and myeloperoxidase (MPO) levels by classical and automated multiplex microbead assay analysis in one reaction environment.

PR3-ANCA		CytoBead			C-ANCA		CytoBead		
		positive	negative	N			positive	negative	N
**ELISA**	Positive	98	25	123	**Classical IIF**	positive	80	9	89
	Negative	18	451	469		negative	10	493	503
	N	116	476	592		N	90	502	592

MPO-ANCA		CytoBead			P-ANCA		CytoBead		
		positive	negative	N			positive	negative	N
**ELISA**	Positive	25	4	29	**Classical IIF**	positive	75	1	76
	Negative	14	549	563		negative	2	514	516
	N	39	553	592		N	77	515	592

		CytoBead (MIA or IIF)					CytoBead (MIA and IIF)		
		positive	negative	N			positive	negative	N
**Classical testing**	Positive	249	9	258	**Classical testing**	positive	116	19	457
**(ELISA or IIF)**	Negative	25	309	334	**(ELISA and IIF)**	negative	16	441	135
	N	274	318	592		N	460	132	592

PR3 ANCA		CytoBead (PR3 ANCA MIA and C-ANCA IIF)			MPO ANCA		CytoBead (MPO ANCA MIA and P-ANCA IIF)		
		positive	negative	N			positive	negative	N
**Classical testing**	Positive	68	7	75	**Classical testing**	positive	24	2	26
**(PR3 ANCA ELISA and C-ANCA IIF)**	Negative	1	516	517	**(MPO ANCA ELISA and P-ANCA IIF)**	negative	4	562	566
	N	69	523	592		N	28	564	592

PR3- and MPO-ANCA were determined by enzyme-linked immunosorbent assay (ELISA) and multiplex CytoBead microbead assay in 118 patients with ANCA-associated vasculitis, 300 with autoimmune and gastrointestinal diseases, 49 with infectious disorders, and 125 blood donors (BD).

The CytoBead technique determined one C-ANCA and one P-ANCA positives more in patients with GPA and MPA, respectively, compared with the classical method.

However, according to McNemar's test, the differences between classical testing and CytoBead analysis were not significant for PR3-ANCA, P-ANCA, and C-ANCA (1.18%, 95% CI: −1.14%–3.34%; 0.17%, 95% CI: −0.41%–0.50%; 0.34%, 95% CI: −1.25%–1.82%; p<0.05, respectively).

In contrast, MPO-ANCA demonstrated a significant difference for both methods (1.69%, 95% CI: 0.14%–2.65%; p = 0.031). Whereas there was no significant difference for positive MPO-ANCA findings obtained by both methods in controls, a tendency for a higher prevalence of positive MPO-ANCA detected by CytoBead microbead immunoassay (35/118, 29.7%) compared to those by EIA (23/118, 19.5%) was found in patients with ANCA-associated vasculitis. The CytoBead microbead immunoassay detected significantly more MPO-ANCA positives in patients with GPA in contrast to the classical ELISA (11/90, 12.2% *vs* 3/90. 3.3%, p = 0.048).

For the serological diagnosis of ANCA-associated vasculitis, a positive ANCA finding by IIF should be confirmed by a positive PR3- or MPO-ANCA result. Thus, we compared the prevalences of confirmed positive ANCA findings by classical testing (IIF and ELISA positive) with multiplex CytoBead analysis (IIF and microbead immunoassay positive) resulting in a very good agreement for both techniques (κ = 0.831, 95% CI: 0.777–0.885). McNemars test did not reveal a significant difference for confirmed positive ANCA findings obtained by classical and multiplex analysis (0.51%, 95% CI: −1.58%–2.50%; p = 0.735).

Furthermore, we compared the prevalences of positive C-ANCA findings by classical IIF testing confirmed by PR3 ANCA ELISA with respective multiplex CytoBead analysis (PR3 ANCA IIF and PR3 ANCA microbead immunoassay positive). There was a very good agreement for both techniques (κ = 0.937, 95% CI: 0.893–0.980) and McNemar's test did not reveal a significant difference (1.01%, 95% CI: −0.07%–1.34%; p = 0.070). The respective comparison of the prevalences for positive P-ANCA findings by classical IIF testing confirmed by MPO ANCA ELISA with multiplex CytoBead analysis (MPO ANCA IIF and MPO ANCA microbead immunoassay positive) revealed also a very good agreement for both techniques (κ = 0.884, 95% CI: 0.792–0.976). McNemar's test did not demonstrate a significant difference (0.34%, 95% CI: −0.56%–0.92%; p = 0.688).

### Comparison of ANCA levels determined by classical and multiplex CytoBead assays

**Figure 3:**
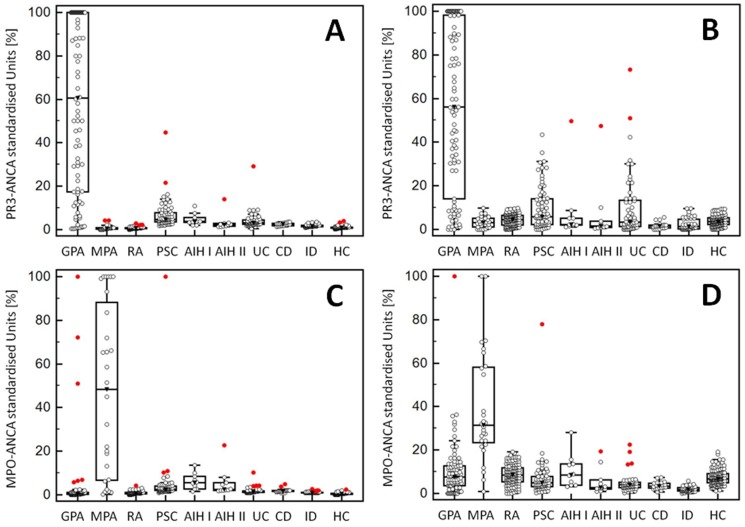
Anti-neutrophil cytoplasmic antibodies (ANCA) against proteinase 3 (PR3) and myeloperoxidase (MPO) levels by classical and automated multiplex microbead assay analysis in one reaction environment. PR3- and MPO-ANCA were determined by enzyme-linked immunosorbent assay (ELISA) (A, C, respectively) and multiplex CytoBead microbead assay (B, D, respectively) in 118 patients with ANCA-associated vasculitis, 300 with autoimmune and gastrointestinal diseases, 49 with infectious disorders, and 125 blood donors (BD). (Data are displayed in Box-and-Whisker plots with *far out* values, defined as values that are smaller than the lower quartile minus 3 times the interquartile range, or larger than the upper quartile plus 3 times the interquartile range, displayed as red circles.). AIH, autoimmune hepatitis; CD, Crohn's disease; GPA, granulomatosis with polyangiitis; ID, infectious diseases; MPA, microscopic polyangiitis; PSC, primary sclerosing cholangitis; RA, rheumatoid arthritis; UC, ulcerative colitis.

**Figure 4:**
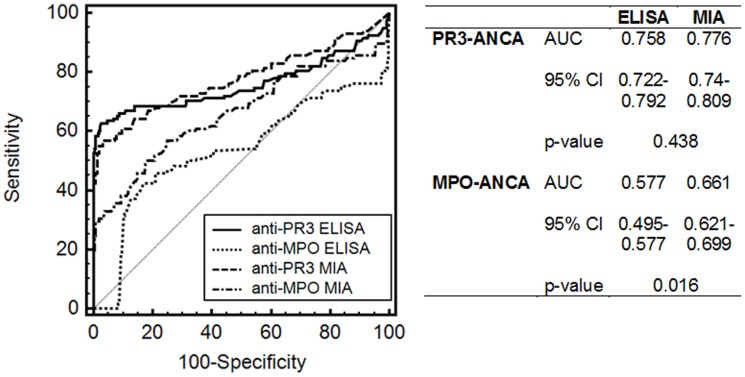
Receiver-operating characteristic curve analysis of anti-neutrophil cytoplasmic antibodies (ANCA) against proteinase 3 (PR3) and myeloperoxidase (MPO) levels by classical and automated multiplex microbead assay testing. PR3- and MPO-ANCA were determined by enzyme-linked immunosorbent assay (ELISA) and multiplex CytoBead microbead assay (MIA) in 118 patients with ANCA-associated vasculitis as disease criterion and in 300 patients with autoimmune and gastrointestinal diseases, 49 with infectious disorders, and 125 blood donors (BD) as control criterion. MPO-ANCA detected by MIA demonstrated a significantly higher AUC compared with those determined by ELISA. AUC, area under the curve; CI, confidence interval.

For the sake of comparison of ANCA levels by classical and multiplex testing, concentrations obtained by the different PR3- and MPO-ANCA assays were harmonized by standardizing values to the cut-offs of the respective assays and reporting them in % (Fig. 3). Standardized data of classical ELISA and multiplex testing by CytoBead microbead immunoassay were subjected to ROC curve analysis using 118 samples of patients with ANCA-associated vasculitis as disease criterion for ANCA *vs* 474 controls (Fig. 4). The AUC for PR3-ANCA by ELISA and microbead immunoassay did not demonstrate a statistical difference (p>0.05). In contrast, there was a significant higher AUC for MPO-ANCA levels by microbead immunoassay compared with those by ELISA (p = 0.016).

### Automated endpoint-ANCA titer evaluation

Employing a test set of 34 sera from patients with GPA (n = 8), MPA (n = 10), RA (n = 6), and BD (n = 10), respective MFI values obtained by 1 to 20 diluted samples by CytoBead technology were compared with classical ANCA-endpoint titers determined by serial dilution in classical IIF. The resulting interdependence of classical P-ANCA and C-ANCA endpoint titers with quantitative MFI obtained by digital IIF were used to establish an automated method for the determination of endpoint ANCA titers in one dilution. Inter-rater agreement statistics revealed a very good agreement comparing both methods for endpoint ANCA titer analysis including all data pairs of titers equal or higher than 10 and combining titers equal or higher than 320 in a 6×6 frequency table (weighted κ = 0.985, 95% CI 0.980–0.991). In routine IIF autoantibody testing, a difference of one titer is not considered significant [31]. Accordingly, automated endpoint ANCA titer analysis of 586 (99.0%) out of the 592 samples investigated did not reveal different titers compared to those detected by the classical method (Table 4). Only 6 (0.1%) sera demonstrated differences of more than one ANCA titer level.

**Table 4:** Comparison of classical with automated anti-neutrophil cytoplasmic antibody (ANCA) endpoint-titer analysis.

Classical (1/titer)										Automated (1/titer)	
	= <10	20	40	80	160	320	640	1280	2560	5120	N
** = <10**	402 (99.3%)	2 (0.5%)	1 (0.2%)	0	0	0	0	0	0	0	405
**20**	8 (12.3%)	55 (84.6%)	1 (1.5%)	1 (1.5%)	0	0	0	0	0	0	65
**40**	0	6 (27.3%)	13 (59.1%)	3 (13.6%)	0	0	0	0	0	0	22
**80**	0	0	5 (26.3%)	12 (63.2%)	2 (10.5%)	0	0	0	0	0	19
**160**	0	0	0	4 (33.3%)	6 (50.0%)	1 (8.3%)	0	1 (8.3%)	0	0	12
**320**	0	0	0	0	6 (24.0%)	12 (48.0%)	7 (28.0%)	0	0	0	25
**640**	0	0	0	0	2 (8.3%)	6 (25.0%)	11 (45.8%)	5 (20.8%)	0	0	24
**1280**	0	0	0	0	0	1 (7.7%)	4 (30.8%)	7 (53.8%)	1 (7.7%)	0	13
**2560**	0	0	0	0	0	0	0	0	1 (50.0%)	1 (50.0%)	2
**5120**	0	0	0	0	0	0	0	0	2 (40.0%)	3 (60.0%)	5
	410	63	20	20	16	20	22	13	4	4	592

ANCA endpoint titers were determined by serial dilution of the 592 samples included in the study by classical indirect immunofluorescence (IIF) and compared to those detected by automated CytoBead IIF on the digital IIF interpretation system Aklides using a 1 to 20 serum dilution only.

## Discussion

For more than 25 years, ANCA serology has been an essential diagnostic tool for the differential diagnosis of vasculitic disorders and IIF is still considered the gold standard for ANCA screening [1], [7], [13]. However, so called obligatory second-line testing to confirm ANCA reactivity by molecular solid-phase immunoassays have been recommended for various reasons [32]. Indeed, IIF is the simplest multiparametric test available allowing the contemporary sensitive detection of C- and P-ANCA. However, its specificity for GPA is obviously lower than that of the antigen-specific PR3-ANCA assays. Thus, the combination of both IIF and antigen-specific assays was confirmed in several studies to be the optimal strategy for ANCA detection [33].

Currently, such tests such as ELISAs, microbead-based or line immunoassays are well established [13], [34], [35]. Their over the past years continuously improved performance, particularly regarding the analysis of PR3-ANCA, have questioned the usefulness of IIF for ANCA testing [24], [25], [36]. Indeed, the need to run two different assay techniques in the recommended two-tier algorithm increases the workload in an already limited in capacity autoimmune laboratory. However, IIF seems to be an indispensable technique in autoimmune diagnostics due to its unsurpassed sensitivity [37]. Apart from ANCA testing, this has been also decisively demonstrated for the assessment of antinuclear antibodies as confirmed by other groups [38], [39].

Hence, combination of the advantages of IIF regarding cell-based assays and its potential for multiplexing by microbead immunoassay within on reaction environment could revolutionize autoimmune diagnostics [15], [40]. Indeed, combining screening and confirmatory testing for disease-specific autoantibodies will generate many benefits ranging from shorter hands-on times, better reproducibility of results to more cost-effectiveness in particular for larger series of samples due the opportunity of using automation and modern data management. We and others could already proved the usefulness of automated ethN-based ANCA testing employing digital immunofluorescence and pattern recognition on novel automated IIF interpretation systems such as Aklides [26], [41]–[44]. Furthermore, we have shown the usefulness of this new IIF technique for multiplexing analysis of autoantibodies [14], [15], [45]. Therefore, we attempted to combine both approaches for effective ANCA testing by IIF in one test environment and to develop additionally an automated interpretation method for the simultaneous pattern interpretation of P- as well as C-ANCA on the one hand and quantitative assessment of PR3- and MPO-ANCA on the other. An earlier attempt by the so called mosaic technique employing several tissue- and cell-based assay sets in one reaction environment did not provide quantitative ANCA interpretation [16].

We could demonstrate a very good to good agreement for P- and C-ANCA as well as PR3- and MPO-ANCA testing by classical and novel multiplex CytoBead analysis. As a matter of fact, the prevalences of positive ANCA confirmed by PR3- or MPO-ANCA showed a very good agreement for both methods. To the best of our knowledge, this is the first report of a combined quantitative screening and confirmatory testing for the serology of ANCA-associated vasculitis. The use of a lower dilution (1 to 20) for PR3- and MPO-ANCA analysis by microbead immunoassay within the reaction environment of the CytoBead technology did not result in a poorer assay performance compared with even third-generation assays for PR3-ANCA. The lower dilution seems to provide a better reaction environment resulting in higher sensitivity mainly for MPO-ANCA. We detected a significantly higher MPO-ANCA prevalence in patients with GPA by the microbead immunoassay compared with ELISA. However, this elevated prevalence of MPO-ANCA could be due to false positive results and needs to be confirmed by further studies. Furthermore, the CytoBead analysis has a greater dynamic range by employing fluorescence instead of optical density measurement in ELISA subjected to the Lambert–Beer law.

The combination of IIF and autoantigen-specific microbead immunoassay resulted in an improvement of the specificity of ANCA testing. In particular patients with RA and AIH type 1 demonstrated a high prevalence of ANCA on ethN not confirmed by molecular PR3- or MPO-ANCA analysis. This phenomenon could be observed for classical as well as multiplex testing in this study and classical ANCA particularly atypical ANCA on ethN have been found in various other diseases than ANCA-associated vasculitis [11], [46]–[48].

The different median ages of the patient cohorts could have an influence on the ANCA assessment. Elevated titers of antinuclear antibodies have been reported in aging individuals leading to a lower specificity of antinuclear antibody testing regarding this population. Since antinuclear antibody positivity could lead to false positive P-ANCA results, a lower false positive rate could be expected in the control groups with lower median ages such as AIH 1, AIH 2, CD, PSC, and UC. However, this was not the case in this study except for patients suffering from UC and PSC regarding PR3-ANCA in particular. Furthermore, our data confirmed recent data of PR3-ANCA positive patients suffering from UC and PSC detected by sensitive assay techniques [49], [50]. Thus, PR3-ANCA might be even proposed as diagnostic parameter for these clinical entities [51]. However, the majority of positive ANCA detected by ethN-based IIF employing both classical and automated IIF were not confirmed by PR3- or MPO-ANCA in these patient cohorts hinting to the presence of other neutrophilic autoantigenic targets.

Quantitative PR3- and MPO-ANCA analysis by multiplex CytoBead technology was at least equal or better compared to classical ELISA testing according to ROC curve analysis. Furthermore, automated endpoint ANCA titer analysis by only one serum dilution using automated IIF interpretation demonstrated a very good agreement with the classical one. Damoiseaux and colleagues could also demonstrate efficient endpoint ANCA titer analysis without serial dilution of samples using digital IIF [51], [52]. We and others have shown the usefulness of automated endpoint titer analysis for other autoantibodies such as ANA [20], [21], [43], [44]. Thus, automated IIF combining screening and confirmatory ANCA analysis simultaneously in one reaction environment appears to be a unique opportunity to replace the time-consuming classical two-tier ANCA testing by a one-step analysis. This is especially important for the emergency diagnostics of rapid progressive glomerulonephritis as an oligosymptomatic manifestation of ANCA-associated vasculitis.

## Conclusions

The CytoBead technology combining screening and confirmatory PR3- and MPO-ANCA testing simultaneously is an alternative to the conventional two-tier ANCA analysis algorithm, which comprises the screening on ethN and confirmation with molecular solid-phase immunoassays. It can be employed for the sensitive and specific detection of ANCA in patients with ANCA-associated vasculitides and probably in patients with UC and PSC as shown elsewhere previously.

The use of digital IIF interpretation systems provides the opportunity to perform automated and standardized quantitative ANCA testing which meets with the demand of modern autoimmune diagnostics in particular for emergency ANCA analysis.

The novel CytoBead technology enables the simultaneous detection of autoantibodies by cell- and microbead-based immunoassays in one reaction environment and, thus, represents an ideal platform for multiplexing of other autoimmune disease-specific antibodies.
