# Correction: Simultaneous Automated Screening and Confirmatory Testing for Vasculitis-Specific ANCA

**DOI:** 10.1371/journal.pone.0120626

**Published:** 2015-03-12

**Authors:** The PLOS ONE Staff

The second affiliation for the 9th author is not indicated. Maria Orietta Borghi is also affiliated with: IRCCS Istituto Auxologico Italiano, Milan, Italy.
