# Correction: Simultaneous Automated Screening and Confirmatory Testing for Vasculitis-Specific ANCA

**DOI:** 10.1371/journal.pone.0127933

**Published:** 2015-05-13

**Authors:** Mandy Sowa, Kai Grossmann, Ilka Knütter, Rico Hiemann, Nadja Röber, Ursula Anderer, Elena Csernok, Dimitrios P. Bogdanos, Maria Orietta Borghi, Pier Luigi Meroni, Peter Schierack, Dirk Reinhold, Karsten Conrad, Dirk Roggenbuck

The second affiliation for the tenth author is not indicated. Pier Luigi Meroni is also affiliated with: IRCCS Istituto Auxologico Italiano, Milan, Italy.

## References

[1] Sowa M, Grossmann K, Knütter I, Hiemann R, Röber N, Anderer U, et al (2014) Simultaneous Automated Screening and Confirmatory Testing for Vasculitis-Specific ANCA. PLoS ONE 9(9): e107743 doi:10.1371/journal.pone.0107743 25225805 10.1371/journal.pone.0107743 PMC4166465

